# Salpingo-oophorectomy versus cystectomy in patients with borderline ovarian tumors: a systemic review and meta-analysis on postoperative recurrence and fertility

**DOI:** 10.1186/s12957-021-02241-2

**Published:** 2021-04-21

**Authors:** Peng Wang, Lei Fang

**Affiliations:** Beijing Obstertrics and Gynecology Hospital, Capital Medical University, No. 251 Yao Jiayuan Road, Chaoyang District, Beijing, 100026 China

**Keywords:** Salpingo-oophorectomy, Cystectomy, Borderline ovarian tumors, Fertility, Meta-analysis

## Abstract

**Background:**

To compare the postoperative recurrence and fertility in patients with borderline ovarian tumors (BOTs) who underwent different surgical procedures: salpingo-oophorectomy versus cystectomy.

**Methods:**

Potentially relevant literature from inception to Nov. 06, 2020, were retrieved in databases including Cochrane Library, EMBASE (Ovid), and MEDLINE (Pubmed). We applied the keywords “fertility-sparing surgery,” or “conservative surgery,” or “cystectomy,” or “salpingo-oophorectomy,” or “oophorectomy,” or “adnexectomy,” or “borderline ovarian tumor” for literate searching. Systemic reviews and meta-analyses were performed on the postoperative recurrence rates and pregnancy rates between patients receiving the two different surgical methods. Begger’s methods, Egger’s methods, and funnel plot were used to evaluate the publication bias.

**Result:**

Among the sixteen eligible studies, the risk of recurrence was evaluated in all studies, and eight studies assessed the postoperative pregnancy rates in the BOT patients. A total of 1839 cases with borderline ovarian tumors were included, in which 697 patients (37.9%) received unilateral salpingo-oophorectomy and 1142 patients (62.1%) underwent unilateral/bilateral cystectomy. Meta-analyses showed that BOT patients with unilateral/bilateral cystectomy had significantly higher recurrence risk (OR=2.02, 95% CI: 1.59-2.57) compared with those receiving unilateral salpingo-oophorectomy. Pooled analysis of four studies further confirmed the higher risk of recurrence in patients with cystectomy (HR=2.00, 95% CI: 1.11-3.58). In addition, no significant difference in postoperative pregnancy rate was found between patients with the two different surgical procedures (OR=0.92, 95% CI: 0.60-1.42).

**Conclusion:**

Compared with the unilateral/bilateral cystectomy, the unilateral salpingo-oophorectomy significantly reduces the risk of postoperative recurrence in patients with BOT, and it does not reduce the pregnancy of patients after surgery.

**Trial registration:**

PROSPERO CRD42021238177

**Supplementary Information:**

The online version contains supplementary material available at 10.1186/s12957-021-02241-2.

## Introduction

Borderline ovarian tumors (BOTs) refer to the tumor of the ovarian with a property between benign and malignant masses, which was first discovered by Tayor in 1929 [1]. It was estimated that about 2.5 to 5.5 per 100,000 women were diagnosed as BOTs every year [2, 3]. According to the classification proposed by the World Health Organization, BOTs are an independent class of ovarian tumors distinguished from epithelial ovarian tumors in clinical and pathological characteristics [4], with an increasing trend of incidence year by year [5]. For the treatment of BOT, surgery is the preferred method for patients at early stages though debates are always exited for different procedures [6]. The last few decades witnessed the rapid development of nanotechnology, a promising method for patients with BOT. This strategy applied particles at nano-levels to deliver drugs to the tumors for local treatment. These particles include nanoparticles, nano-micelles, nanocapsules, and liposomes, which have obtained satisfied effects for the diagnosis and treatment of cancers [7]. BOTs are more commonly discovered in women at premenopausal status, especially before 40 years old [8, 9]. At this special life stage, many diagnosed women express strong expectation of preserving fertility. Therefore, a fertility-sparing treatment (FST) is particularly important for these patients.

Previous studies showed that FST can be tolerated in BOT patients and the ability of pregnancy was persevered [10, 11], while other studies revealed that the risk of recurrence, or even death, may be increased in patients receiving FST [12–14]. As we known, FST has various surgical procedure types including salpingo-oophorectomy and cystectomy. Currently, there is no consensus on the selection of preferred surgical procedures for patients with BOT. Some researchers believed that salpingo-oophorectomy was showed a lower recurrence rate, thereby tending to select salpingo-oophorectomy in these patients, while other surgeons proposed that resection of one ovary may lead to declined possibility of pregnancy, thus advocating the cystectomy [15]. Given these inconsistent results and opinions, it is needed to comprehensively summarize and analyze the current evidences on this topic, thereby clarifying the advantages and disadvantages of these two surgical methods.

In order to assess the postoperative recurrence and fertility in patients with BOTs who underwent salpingo-oophorectomy or cystectomy, we conducted this systemic review and meta-analysis for the currently available studies. It is hoped to provide clinical evidences for surgeons to select better surgical procedures for BOT patients.

## Methods

This systemic review and meta-analysis was performed in strict accordance with the Preferred Reporting Items for Systematic Reviews and Meta Analyses (PRISMA) [16].

### Literature search

All potentially relevant literatures from inception to Nov. 06, 2020, were searched in the databases including Cochrane Library, EMBASE (Ovid), and MEDLINE (Pubmed) [17]. The MeSH words “fertility,” or “postoperative recurrence,” or “conservative surger,” or “cystectomy,” or “salpingo-oophorectomy,” or “oophorectomy,” or “adnexectomy,” or “borderline ovarian tumor,” or “systemic review,” or “meta-analysis” were used for literate retrieval.

### Eligibility of studies and data extraction

Two authors independently screened the articles. The preliminarily searched literature was firstly screen with tittle, and then selected by reading the abstract. After a comprehensive review of the full text of selected literature, articles that met the inclusion and exclusion criteria were finally included for data extraction. If there was inconsistency about article inclusion, it was determined by an in-depth discussion of the two authors. Only the study with the largest sample size was included if there were many studies with the same population for investigation. The inclusion criteria for eligible literature were as follows: (1) all patients were pathologically confirmed as BOTs after surgery; (2) studies comparing the oncological outcomes and the postoperative pregnancy of two FSTs, that were the unilateral/bilateral cystectomy versus unilateral salpingo-oophorectomy, in patients with BOTs; (3) studies with sample size ≥ 30. Exclusion criteria were as follows: (1) studies that did not provide data of relevant clinical outcomes; (2) studies with incomplete data; (3) reviews, meta-analysis, case reports, meeting abstracts, and other types of articles that were inappropriate for this study. A predesigned form was used for data extraction of the studies. Here, we collected the author name, country, study period, age, sample size, surgical procedures, stage, follow-up, recurrence, 5-year disease-free survival, interval to recurrence, pregnancy, time to pregnancy, and risk of bias.

### Main outcomes

In this study, the main clinical outcomes for meta-analysis include the rate of postoperative recurrence and the rate of pregnancy in BOT patients who underwent unilateral/bilateral cystectomy or unilateral salpingo-oophorectomy.

### Assessment of literature quality

To evaluate the quality of the included literature, we systemically collected the parameters of each study such as country, study period, sample size, follow-up, and so on. The risk of bias in non-randomized studies of interventions (ROBINS-I) was used for assessing the risk of bias. The publication bias was assessed by Begger’s method, Egger’s method, or funnel plot.

### Data analyses

All the statistical analyses were conducted with the Stata 14.0 software. In order to compare the postoperative recurrence and pregnancy between unilateral/bilateral cystectomy and unilateral salpingo-oophorectomy, pooled odds ratios (ORs) or hazard ratios (HRs) with the 95% confidence interval were calculated in meta-analyses. The heterogeneity of studies was assessed by the *I*^*2*^ test or Cochran *Q* test. *I*^*2*^ > 50% or *P* < 0.1 indicated the existence of significant heterogeneity, and the random-effects model was applied for meta-analysis. In other situations, the fixed random-effects model was used.

## Results

### General characteristics of included studies

After the preliminary retrieval with the keywords in the databases, 425 articles with potential relevance were obtained. A total of 380 articles were excluded after reading the title and abstract by the two investigators. For the remaining 45 articles, the full texts were carefully reviewed, and 29 articles (including 5 reviews or meta-analysis, 16 articles with incomplete data or irrelevance, and 8 studies with a sample size less than 40) did not meet the criteria for eligibility. Eventually, 16 studies were included in the data synthesis of meta-analysis (Table 1). All the studies were retrospective cohort studies published between 2001 and 2019. A total of 1839 cases with borderline ovarian tumors were included, in which 697 patients (37.9%) received unilateral salpingo-oophorectomy and 1142 patients (62.1%) underwent unilateral/bilateral cystectomy. The sample sizes of the included studies were from 31 to 535 cases.

**Table 1:** Baseline characteristics of included studies

Study	Country	Study period	Age median (range)	Sample size (Cy vs SO)	Stage	Follow-up median (range)	Recurrence (Cy vs SO, %)	5-DFS (Cy vs SO, %)	Interval to recurrence	Pregnancy (Cy vs SO, %)	Time to pregnancy	Risk
Marchette et al.	Italy	1978-2013	29.8 (25.3-34.4)	535 (264/271)	I-IV	12.4 y (11.8-13.3)	54.7% vs 45.3%	HR: 1.34 (0.98-1.81)	N/A	52.4% vs 47.6%	N/A	M
Tsai et al.	Taiwan	2000-2006	Mean±SD: 40.7±16.5	31 (7/24)	I-III	56.5 mo (12-103)	22.6%	N/A	25.1 mo (10-56)	19.4%	N/A	M
Yinon et al.	Israel	1979-2014	28 (13-44)	62 (22/40)	I	36 mo (7-81)	22.2% vs 27.5%	N/A	23.6 vs 41 mo	22.7% vs 47.5%	42 mo (9-144)	M
Morice et al.	France	1965-1997	Mean±SD: 32±11.4	49 (11/38)	I-III	109 mo (24-300)	36.3% vs 15.1%	N/A	38 mo (1-243)	28.6%	37.5 mo (3-84)	M
Romagnolo et al.	Italy	1992-2004	44 (20-88)	53 (21/32)	I-III	44 mo (6-122)	28% vs 23%	N/A	N/A	15.1%	N/A	H
Poncelet et al.	France	1990-2000	Mean±SD: 30.6±7.8	133 (33/100)	I	19 mo (6-243)	30.3% vs 11%	N/A	N/A	N/A	N/A	M
Chen et al.	China	2003-2010	30.2 (11-49)	122 (75/47)	I-III	Cy: 22.7 mo SO: 48.0 mo	9.3% vs 2.1%	N/A	22.7 vs 48.0 mo	77.3% vs 76.9%	N/A	M
Lee et al.	Korea	1998-2014	Cy: 28 (14-40) SO: 30 (21-38)	108 (19/89)	I	Cy: 25.4 mo SO: 37.4 mo	15.8% vs 3.4%	78.8% vs 95.7%	24 mo	87.5% vs 79.2%	N/A	M
Fang et al.	China	1996-2016	N/A	45 (7/38)	I-III	46.5 mo (13-146)	63% vs24%	HR: 3.30 (1.34-8.14)	27 vs 55 mo	67% vs 69%	N/A	M
Uzan et al.	France	1999-2009	29 (14-65)	119 (69/50)	I	45 mo (12-120)	37.7% vs 24%	N/A	N/A	27%	48 vs 27 mo	M
De Iaco et al.	Italy	1985-2006	45.5 (14-85)	85 (35/50)	I-III	60.5 mo (4-240)	34.3% vs 20.0 %	59.6% vs 78.4%	25.1 mo	N/A	N/A	M
Song et al.	Korea	1997-2009	29 (10-83)	155 (38/117)	I-III	56 mo (0.6-155.9)	13.2% vs 5.9%	N/A	N/A	88.2%	28 mo (8-97)	M
Burgmann et al.	USA	1982-2005	33 (12-95)	190 (47/143)	I	N/A	23% vs 7%	N/A	2.6 vs 4.8 y	N/A	N/A	H
Pektas et al.	Turkey	1999-2009	Mean±SD: 37.4±9.5	50 (14/36)	I-III	61.0±23.2 mo	14.3% vs 2.8%	Mean DFS: 60.7± 23.2	N/A	52.3%	N/A	M
Koskas et al.	France	1997-2004	Mean±SD: 26.5±6.4	31 (12/19)	I	59 mo (12-182)	41.7% vs 5.3%	49.1% vs 94.7%	N/A	41.8 vs 45.9%	N/A	M
Ureyen et al.	Turkey	1990-2014	38.5 (18-74)	71 (23/48)	I-III	57 mo (3-270)	17.4% vs 8.3%	N/A	N/A	N/A	N/A	H

*Cy* cystectomy (unilateral and/or bilateral), *SO* unilateral salpingo-oophorectomy, *mo* months, *DFS* disease-free survival, *HR* hazard ratio, *M* moderate risk, *L* low risk, *H* high risk

### Comparison of postoperative recurrence risk between patients with unilateral salpingo-oophorectomy and unilateral/bilateral cystectomy

**Fig. 1:**
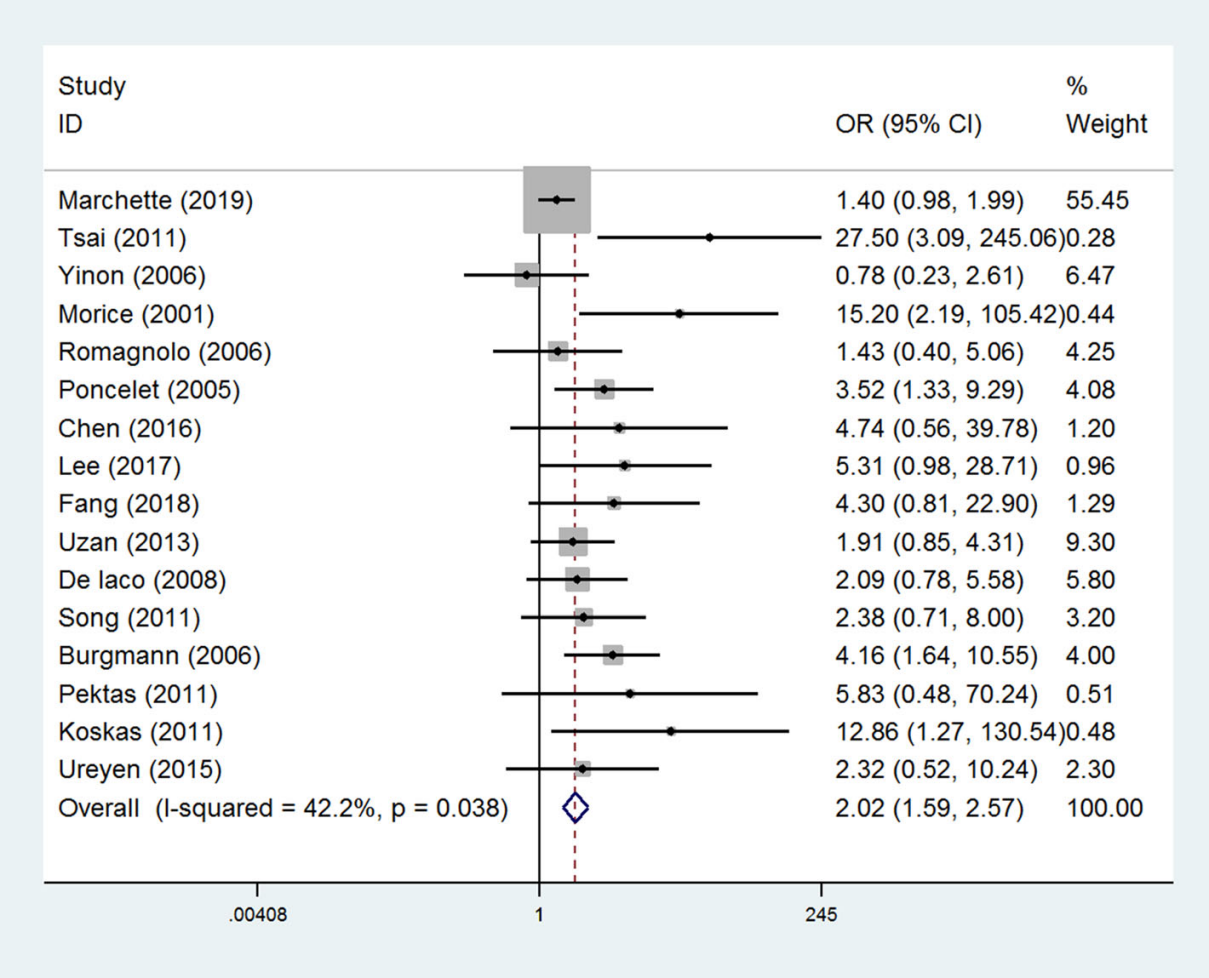
Forest plots of the odds ratios of postoperative recurrence in BOT patients. Sixteen studies compared the risk of BOT recurrence in between patients underwent unilateral salpingo-oophorectomy and unilateral/bilateral cystectomy. The results of the pooled analysis revealed that the rate of tumor relapse was significantly higher in patients receiving unilateral/bilateral cystectomy had a significantly higher risk of tumor relapse

**Fig. 2:**
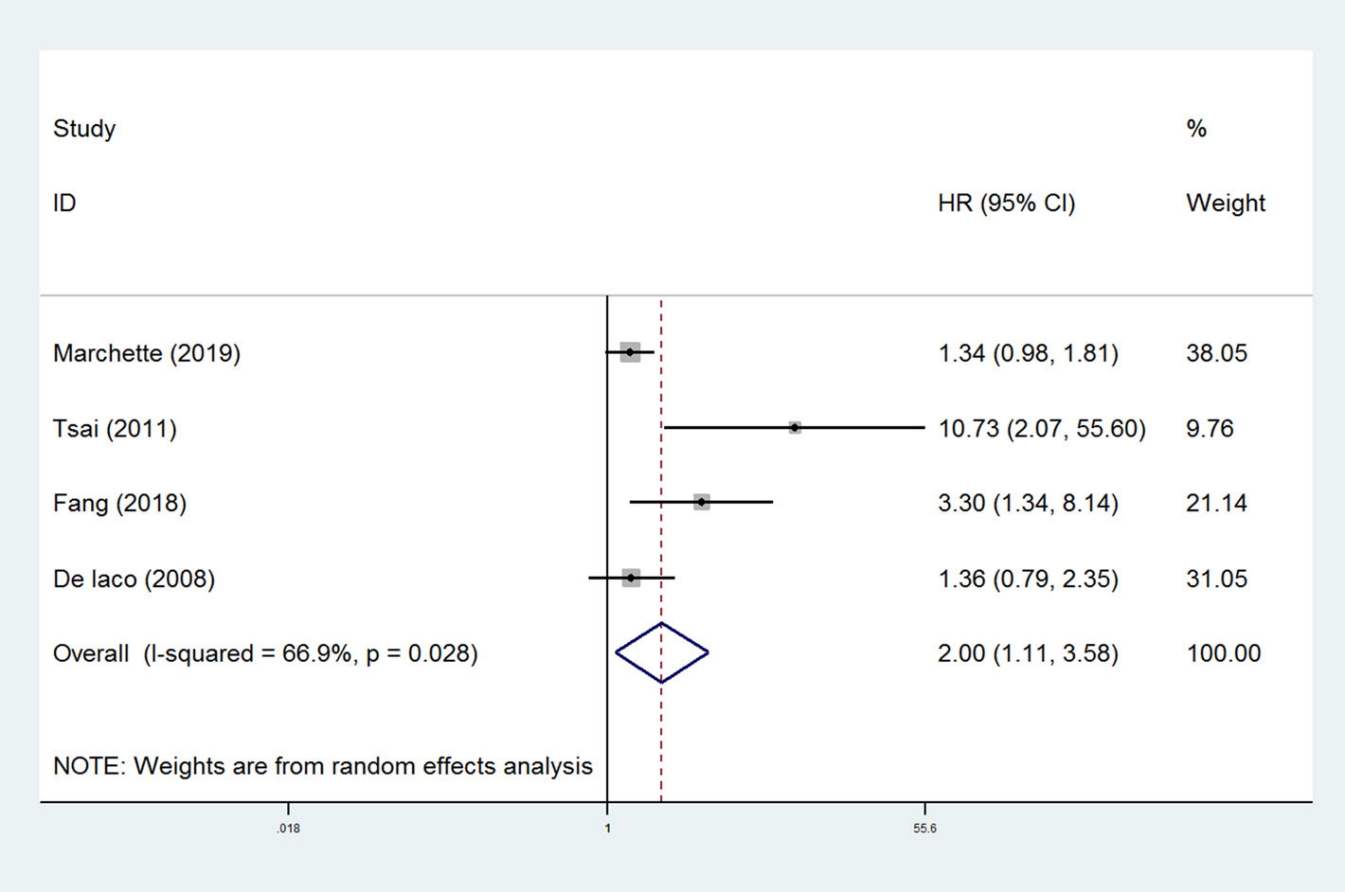
Forest plots of the hazard ratios of postoperative recurrence in BOT patients. Four studies calculated the hazards ratio (HR) and 95% CI of unilateral/bilateral cystectomy for risk of tumor recurrence compared with unilateral salpingo-oophorectomy. Results confirmed that cystectomy was associated with a higher recurrence rate of BOT

Sixteen studies compared the risk of BOT recurrence between patients underwent unilateral salpingo-oophorectomy and unilateral/bilateral cystectomy (Table 2) [12, 18–32], and the results of the pooled analysis revealed that the rate of tumor relapse was significantly higher in patients receiving unilateral/bilateral cystectomy (OR=2.02, 95% CI: 1.59-2.57, *P* < 0.001, Fig. 1). Begger’s and Egger’s methods were used to evaluate the publication bias of the literature. The plots did not show significant publication bias (Supplementary Figures 1 and 2). A total of four studies calculated the hazards ratio (HR) and 95% CI of unilateral/bilateral cystectomy for tumor recurrence compared with unilateral salpingo-oophorectomy [21, 26–28]. Meta-analysis further confirmed that cystectomy was associated with a higher recurrence rate of BOT (HR=2.00, 95% CI: 1.11-3.58, *P* < 0.001, Fig. 2). A funnel plot was drawn and no significant publication bias was presented (Supplementary Figure 3).

**Table 2:** Subgroup analysis for the comparison of recurrence between salpingo-oophorectomy and cystectomy in borderline ovarian tumors

Subgroup analysis	Studies	Pooled results		Heterogeneity	
		Effect size (95% CI)	***P*** value	***I***^**2**^	***P*** value
Region		OR			
Eastern	5	2.02 (2.21-9.40)	<0.001	0%	0.443
Western	11	1.83 (1.42-2.36)	<0.001	42.3%	0.067
FIGO stage		OR			
I	7	3.16 (1.92-5.20)	<0.001	21.7%	0.264
I-III	9	1.78 (1.35-2.33)	<0.001	43.6%	0.077
Sample size		OR			
≥100	7	1.85 (1.40-2.44)	<0.001	36.3%	0.151
<100	9	2.64 (1.64-4.23)	<0.001	47.1%	0.057
Publication year		OR			
After 2010	10	1.89 (1.42-2.50)	<0.001	41.0%	0.084
Before 2010	6	2.43 (1.56-3.79)	<0.001	47.0%	0.093
Follow-up period		OR			
≥50 months	8	1.84 (1.37-2.47)	<0.001	57.9%	0.020
<50 months	7	2.16 (1.38-3.39)	0.001	7.1%	0.374

### Comparison of postoperative fertility between patients with different surgical procedures

**Fig. 3:**
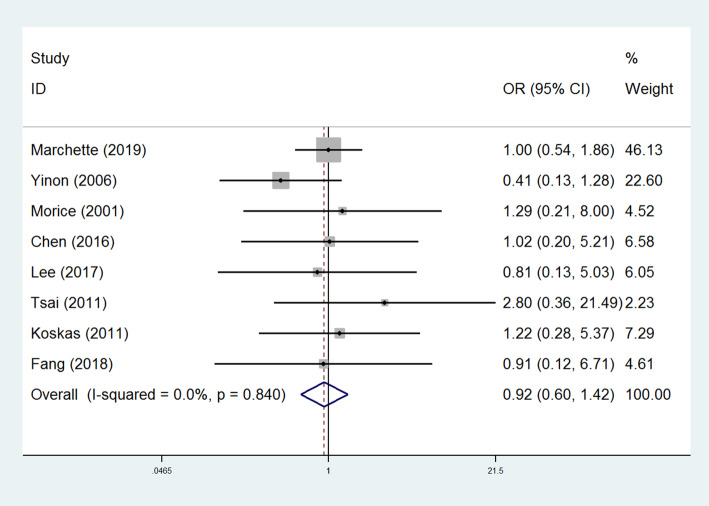
Forest plots of the odds ratios of postoperative pregnancy in BOT patients. Eight studies evaluated the postoperative pregnancy rates of patients after fertility-sparing treatment. A pooled analysis did not reveal a significant difference in the postoperative pregnancy rates between patients in the two groups

Eight studies evaluated the postoperative pregnancy rates of patients after fertility-sparing treatment [20, 21, 23, 25–27, 31, 32]. A pooled analysis did not reveal a significant difference in the postoperative pregnancy rates between patients underwent unilateral/bilateral cystectomy (OR=0.92, 95% CI: 0.60-1.42, *P* > 0.05, Fig. 3), suggesting that the two surgical procedures had similar effects on the postoperative fertility in patients with BOT. No significant publication bias was presented on the funnel plot (Supplementary Figure 4).

## Discussion

In this systemic review and meta-analysis, we found that BOT patients with unilateral/bilateral cystectomy were showed a higher risk of relapse compared with those who underwent unilateral salpingo-oophorectomy. Moreover, there was no significant difference in the postoperative pregnancy rates between patients with the two different surgical procedures. Our study provides valid evidence on the clinical outcomes, as well as effects on pregnancy, of the unilateral/bilateral cystectomy and salpingo-oophorectomy in patients with BOTs. It is of great significance to verify the advantages of salpingo-oophorectomy in clinical practices.

The typical features of BOTs are proliferation of tumor cells and the atypia of the nucleus, while the stroma is not invaded. It is more common in young females and the prognosis is better than that of invasive epithelial ovarian cancers [33]. It was reported that the patients with BOTs diagnosed at childbearing age accounted for about 34% of all the patients [34]. One of the obstacles influencing the prognosis of BOTs is the relapse. It was estimated that the rate of relapse in BOT patients was about 0% to 25%, in which 1% to 3% of the relapse were invasive [35]. In 2007, Silva et al. found that the recurrence of BOTs varied along with follow-up duration, with a 5-year, 10-year, 15-year, and more than 15-year relapse rate of 10%, 19%, 10%, and 5%, respectively [36]. However, one study found that the postoperative pregnancy rate was declined to 34% while the risk of relapse was increased to 38% in BOT patients at late stages [15]. A pooled analysis revealed a relapse rate of 16% including 5 cases of death and 48% of the patients were pregnant after FST [37]. Since the large number of BOT patients at a life stage with the expectation of fertility, treatment methods were increasingly from radical resection to a more conservation strategy [10]. In particular, FST is a method with satisfied safety, and has been widely used in ovarian tumors [1–3]. Even though the FST was already recognized as an important strategy in patients with BOTs, studies for comparing the clinical outcomes and prognosis between different FST subtypes such as cystectomy and salpingo-oophorectomy are rare.

Cystectomy has the advantage of retaining more ovarian tissues, which is proposed to greatly increase the possibility of pregnancy after surgery. One study investigated the effects of four kinds of FSTs including unilateral adnexectomy, unilateral cystectomy, unilateral adnexectomy plus contralateral cystectomy, and bilateral cystectomy in patients with BOTs [20]. Patients in the unilateral cystectomy group had a higher relapse rate (which was increased to 9.3%) and shorter interval of relapse (which was decreased to 22.7 months) compared with those in the unilateral adnexectomy group. Nevertheless, Song and colleagues showed in their study that patients receiving bilateral cystectomy had a significantly higher cumulative rate of pregnancy compared with patients receiving unilateral adnexectomy plus contralateral cystectomy (14/15 versus 9/17) [19]. According to these results, cystectomy can be considered as a method for surgery in patients with extremely strong expectations of pregnancy. Even so, in our study, the pooled analysis showed a significantly higher risk of recurrence in patients with unilateral/bilateral cystectomy compared with salpingo-oophorectomy. More importantly, the rate of postoperative pregnancy was similar between these two surgical approaches. It is preliminarily suggested that salpingo-oophorectomy may be a better choice for BOT patients.

Many previous reports advocated the advantage of salpingo-oophorectomy in lower recurrence rate compared with cystectomy [25, 26, 31]. However, other studies did not detect a significant difference in postoperative relapse rate between patients with cystectomy and salpingo-oophorectomy [20, 24, 32]. Some scholars argued that the main reason for this discrepancy was the difference in the sample size of studies [32], which should be further clarified in future studies with strict design. Our meta-analysis here confirmed that the risk of recurrence was indeed significantly higher in patients with cystectomy than those with salpingo-oophorectomy (OR=2.02, 95% CI, 1.59-2.57). In addition, the subgroup analysis for the four studies, in which HRs were calculated, further supported a higher pooled HR (HR=2.00, 95% CI, 1.11-3.58) for patients who underwent cystectomy compared with those receiving salpingo-oophorectomy. Therefore, our study verified a preference for salpingo-oophorectomy than cystectomy for surgery decision-making in patients with BOTs.

### Limitations

There are some limitations of this meta-analysis. First of all, the included studies were retrospective cohort studies, which limited the strength of evidence levels. Secondly, only 4 studies evaluated the HRs and 95% CI for BOT recurrence and there was curtain heterogeneity among these studies. Further considerations on the source of heterogeneity should be paid attention to when conducting related studies to confirm these results. Thirdly, since the diagnoses were confirmed by pathological examination after surgery, the selection bias was inevitably existed in the included studies. Finally, we did not investigate and compare the survival data between patients who underwent the two different surgical procedures, which should be clarified in further analyses.

## Conclusion

On the basis of our pooled analysis of previous studies, this study confirmed that compared with the unilateral/bilateral cystectomy, the unilateral salpingo-oophorectomy significantly reduces the risk of postoperative recurrence in patients with BOT, and it does not influence the pregnancy of patients after surgery. For BOT patients with the expectation of pregnancy, unilateral salpingo-oophorectomy may be a preferred choice for treatment.

## Supplementary Information

**Additional file 1: Supplementary Figure 1**: Begg’s funnel plot with pseudo 95% confidence limits

**Additional file 2: Supplementary Figure 2**: Egger’s publication bias plot

**Additional file 3: Supplementary Figure 3**: Funnel plot with pseudo 95% confidence limits

**Additional file 4: Supplementary Figure 4**: Funnel plot with pseudo 95% confidence limits

## Data Availability

The current study was based on the results of relevant published studies.
