# Supplementary figures and images for: Salpingo-oophorectomy versus cystectomy in patients with borderline ovarian tumors: a systemic review and meta-analysis on postoperative recurrence and fertility
Source: World J Surg Oncol. 2021 Apr 21;19:132. doi: 10.1186/s12957-021-02241-2 (PMC8061226; doi:10.1186/s12957-021-02241-2)

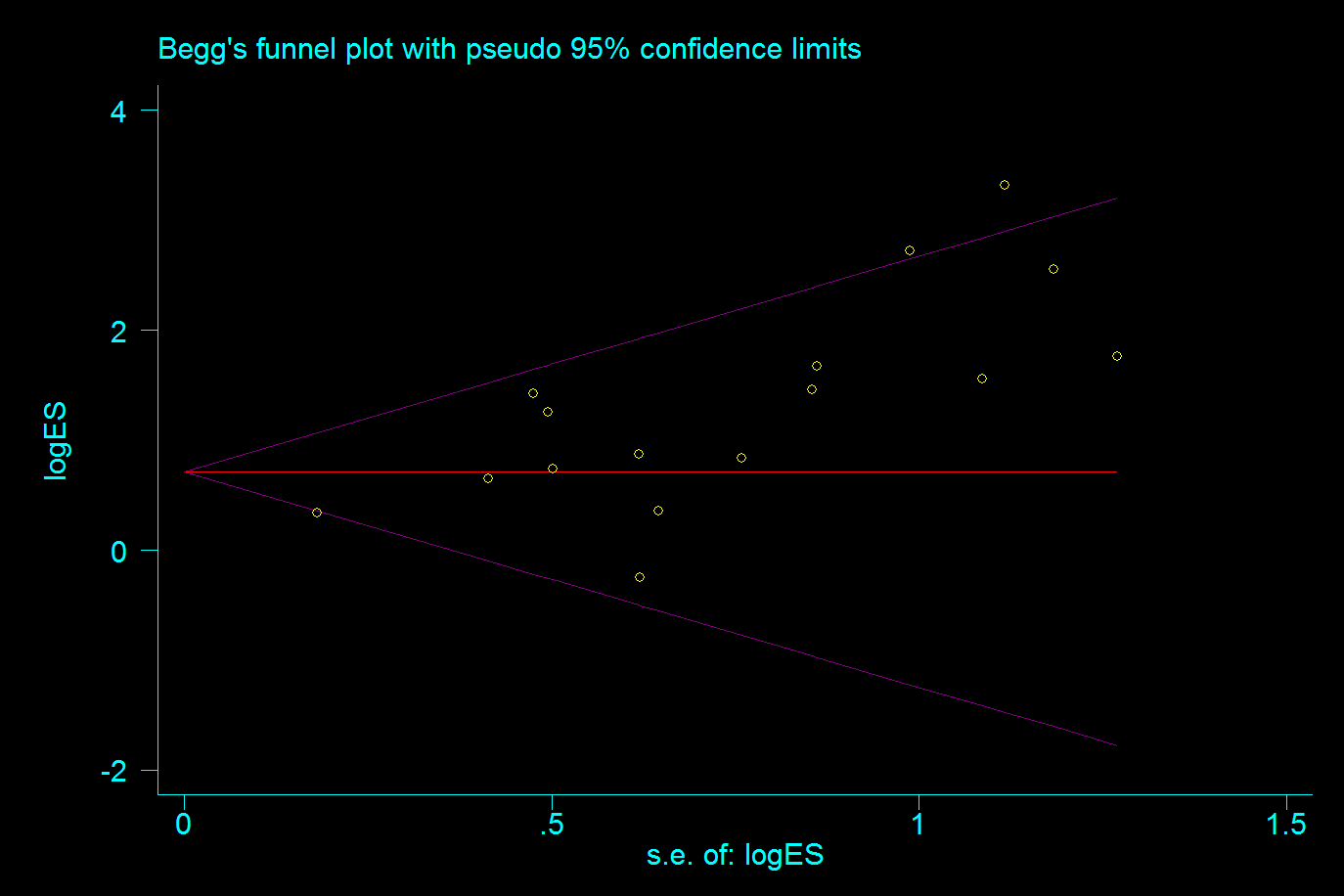

Supplement: Supplementary file 1 — Additional file 1: Supplementary Figure 1: Begg’s funnel plot with pseudo 95% confidence limits [file 12957_2021_2241_MOESM1_ESM.tif]

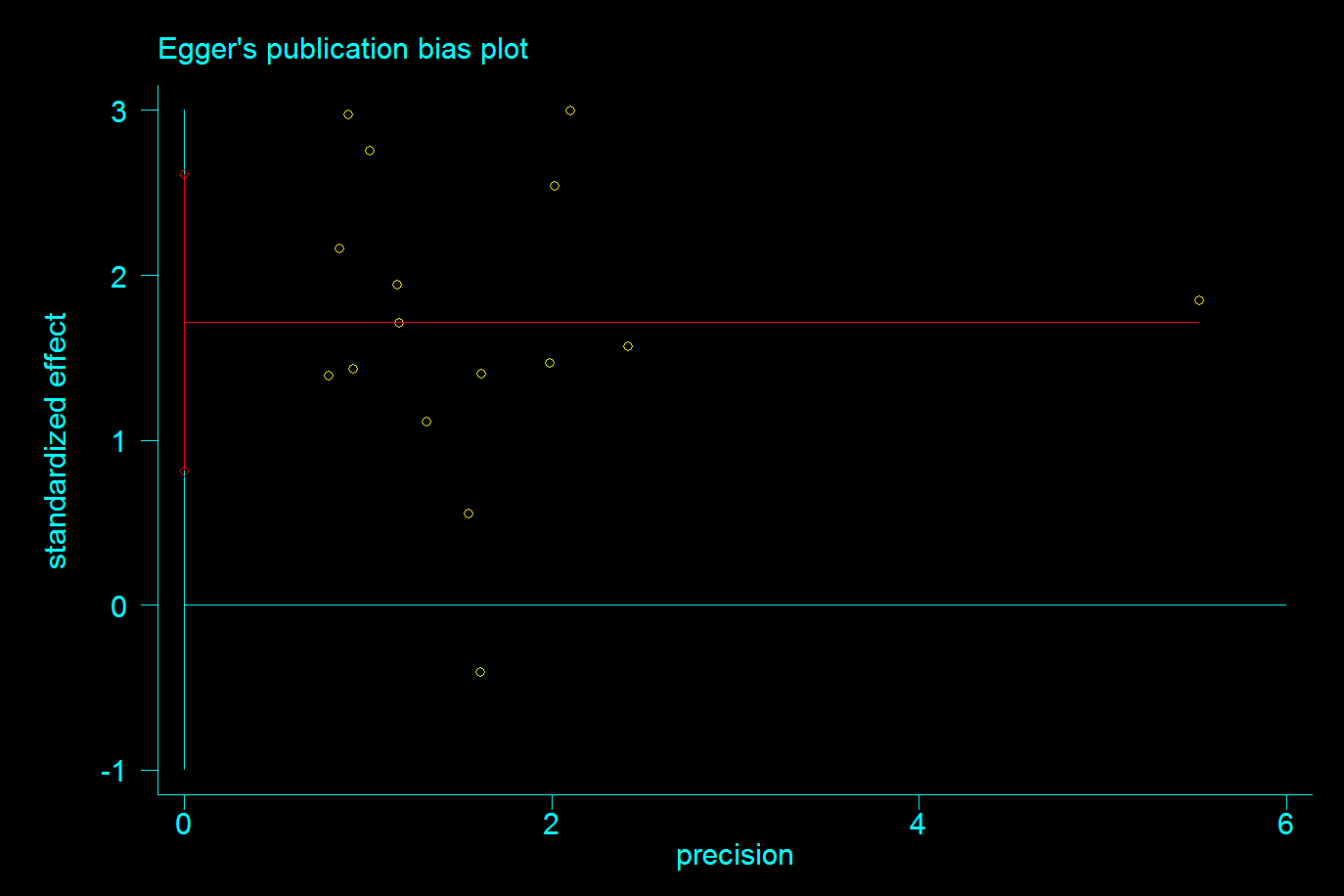

Supplement: Supplementary file 2 — Additional file 2: Supplementary Figure 2: Egger’s publication bias plot [file 12957_2021_2241_MOESM2_ESM.tif]

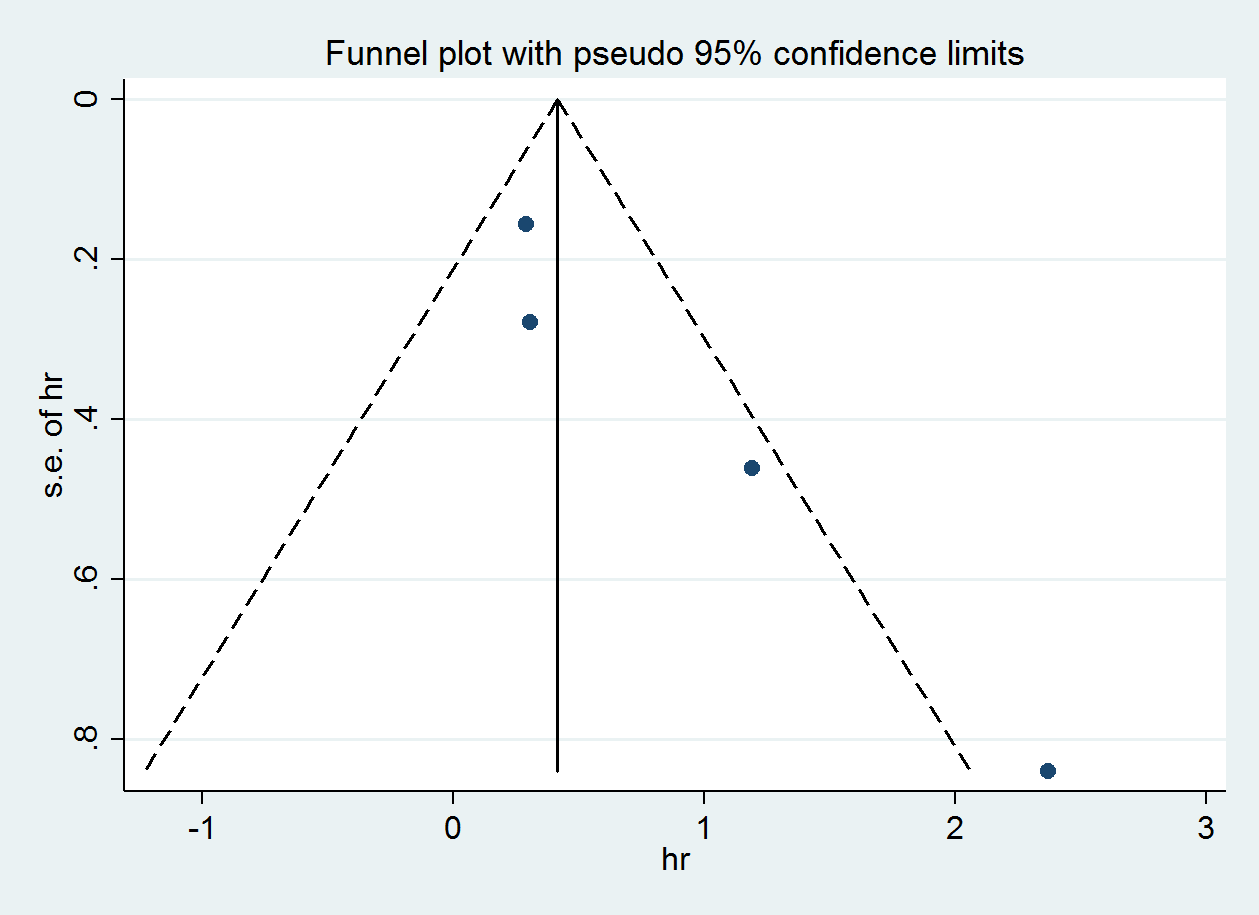

Supplement: Supplementary file 3 — Additional file 3: Supplementary Figure 3: Funnel plot with pseudo 95% confidence limits [file 12957_2021_2241_MOESM3_ESM.tif]

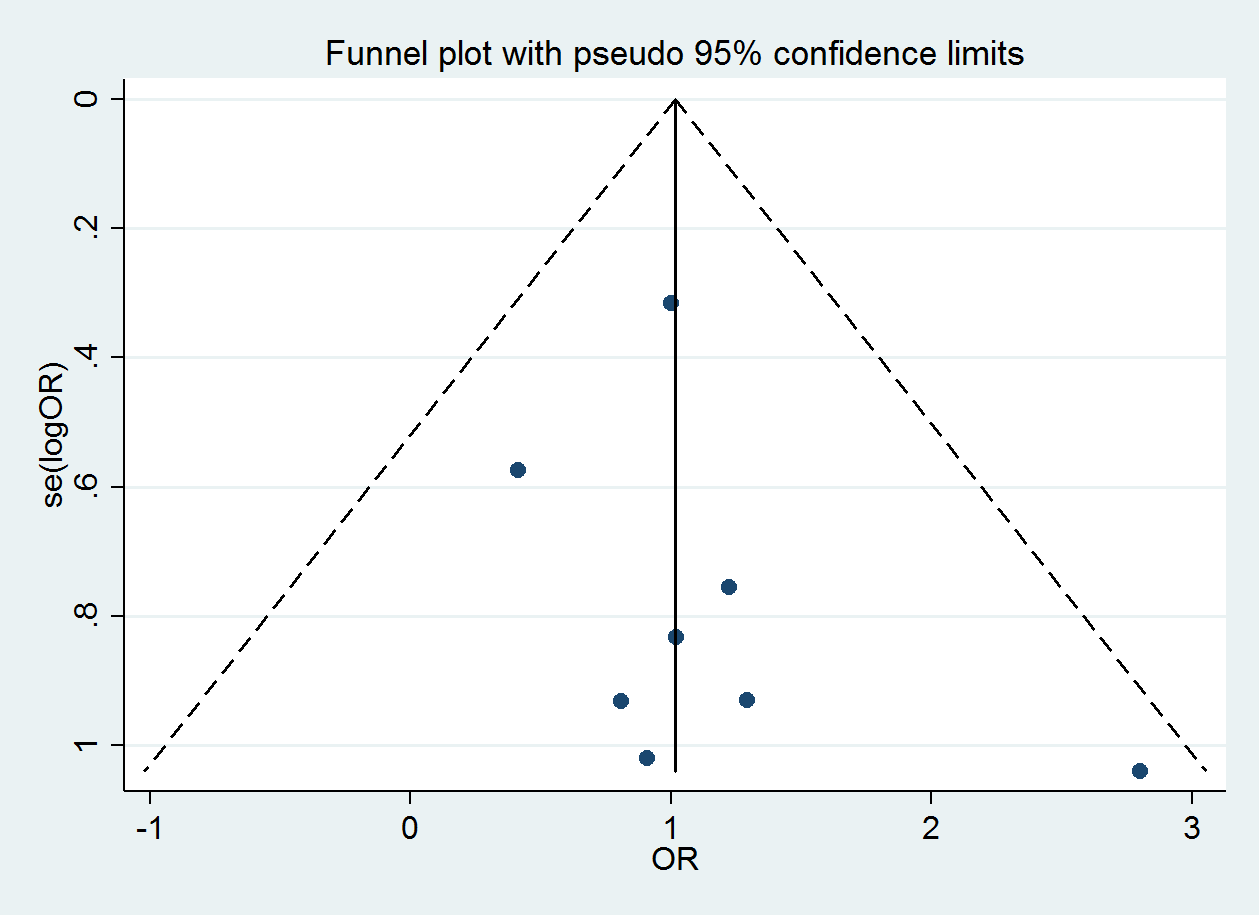

Supplement: Supplementary file 4 — Additional file 4: Supplementary Figure 4: Funnel plot with pseudo 95% confidence limits [file 12957_2021_2241_MOESM4_ESM.tif]
